# The Conditioned Medium of Calcined Tooth Powder Promotes the Osteogenic and Odontogenic Differentiation of Human Dental Pulp Stem Cells via MAPK Signaling Pathways

**DOI:** 10.1155/2019/4793518

**Published:** 2019-03-19

**Authors:** Jintao Wu, Na Li, Yuan Fan, Yanqiu Wang, Yongchun Gu, Zehan Li, Yin Pan, Gobin Romila, Zuomin Zhou, Jinhua Yu

**Affiliations:** ^1^Endodontic Department, School of Stomatology, Nanjing Medical University, 136 Hanzhong Road, Nanjing, Jiangsu 210029, China; ^2^Institute of Stomatology, Nanjing Medical University, 136 Hanzhong Road, Nanjing, Jiangsu 210029, China; ^3^Cenral Lab, First People's Hospital of Wujiang Dist, Suzhou, Jiangsu, China; ^4^Department of Histology and Embryology, Nanjing Medical University, 140 Hanzhong Road, Nanjing, Jiangsu 210029, China

## Abstract

The calcined tooth powder (CTP), a type of allogeneic biomimetic mineralized material, has been confirmed that can promote new bone formation when obtained at high temperature. The aim of this study was to investigate effects of the conditioned medium of calcined tooth powder (CTP-CM) on the osteogenic and odontogenic differentiation of human dental pulp stem cells (hDPSCs) and the underlying mechanisms involved. First, ALP activity assay determined that 200 *μ*g/mL was the optimal concentration of CTP-CM for the following experiments. CTP-CM had no significant effect on the proliferation of hDPSCs as indicated by CCK-8 and FCM analysis. Both the gene and protein (*DSPP*/DSPP, *RUNX*2/RUNX2, *OCN*/OCN, *OSX*/OSX, *OPN*/OPN, *ALP*/ALP, and *COL*-1/COL-1) expression levels increased in the CTP-CM-induced hDPSC group as compared with those in the control group at day 3 or 7, showing the positive regulation of CTP-CM on the osteo/odontogenic differentiation of hDPSCs. Mechanistically, MAPK signaling pathways were activated after the CTP-CM treatment, and the inhibitors targeting MAPK were identified which weakened the effects of CTM-CM on the committed differentiation of hDPSCs. These findings could lead to the creation of stem cell therapies for dental regeneration.

## 1. Introduction

The ultimate aim of tissue engineering is to research and develop biological substitutes, to rescue or/and restore various forms of damage and defect, and to maintain and enhance the function of tissues or organs [1]. Stem cells and biomaterials and the interactions between them are the most important part of the development process of tissue engineering [2]. Over the past decade, scientists from home and abroad become increasingly interested in stem cell research, which became a novel and promising candidate approach for cell-based therapeutic strategies, owing to their intrinsic ability of self-renewal and multilineage differentiation potential [3–5]. There is a considerable amount of data available that stem cells existed in various areas of the human body, such as bone marrow, skin, teeth, heart, liver, skeletal muscle, peripheral blood, and blood vessels [6]. The teeth and the surrounding tissues are an important source of stem cells. Dental stem cells are thought to originate from the cranial neural crest and were documented, successfully isolated, and characterized, including hDPSCs [7], stem cells isolated from apical papilla (SCAPs) [8, 9], periodontal ligament stem cells (hPDLSCs) [10], stem cells from human exfoliated deciduous teeth (SHED), stem cells isolated from dental follicle precursor cells (DFPCs), and stem cells from human gingiva. HDPSCs, which are successfully isolated from human third molars in 2000 for the first time, are increasingly being recognized as a viable cell source for dental regenerative medicine [11, 12]. Research on hDPSCs has become one of the fastest-growing areas in regenerative medicine, mainly because hDPSCs have the following characteristics: they are easy accessible, they share the same origin, the efficiency and success rate of isolating are high, the lifespan of these stem cells is long, and these cells can be safely cryopreserved [13–16]. HDPSCs share similar features with undifferentiated mesenchymal stem cells, which own remarkable self-renewal potential and multilineage differentiation capability [17, 18]. HDPSCs can differentiate into multiple cell lineages including odontoblasts, osteoblasts, chondrocytes, adipocytes, and neurons under defined induction conditions *in vitro* [19–22].

In endodontics treatment, the pulp capping material, which was conventionally used in vital pulp therapy, is calcium hydroxide. However, it is reported that calcium hydroxide is not capable of biologically inducing odontogenic differentiation or accelerating reparative dentin formation [23]. Recently, a novel biomaterial, mineral trioxide aggregate (MTA), has been increasingly widely used in endodontic practice. But disadvantages of MTA—the setting time is long, the material compatibility with other dental materials when layered is not well, and it is difficult to control its consistency—are problems in clinical practice [24]. Autogenous materials, such as autogenous bone, are the most ideal since they enhance bone regeneration without immune response in healing time and can gradually be resorbed [25, 26]. Their biggest shortcomings are that the amount we can harvest is limited. Therefore, to overcome such shortcomings, autogenous tooth bone graft materials obtained at high temperature were examined, showing that their characteristics were similar to those of autogenous bones [25]. The human teeth are mainly made up of hydroxyapatite (HA) [27], whose inorganic components are similar to those of alveolar bone. Teeth are extracted, due to various reasons, such as periodontal diseases, trauma, wisdom teeth, or premolars extracted for orthodontic reasons. When these teeth were burnt at high temperatures and grinded, tooth ash was obtained. After teeth are calcined at high temperature, their organic components, which may cause infections or immune reactions, are destroyed [28]. The remaining ash is inorganic components mainly comprised of HA and TCP [29, 30]. In addition, the proportions of HA and TCP are different when teeth ash are obtained from different teeth types (permanent teeth ash (PTA), deciduous teeth ash (DTA)) or permanent teeth calcined at different temperatures [28]. Previous studies have shown that particulate dentin (tooth ash, tooth particles) has the capacity of osteoconduction. And the main advantage of tooth ash is that it can be resorbable *in vivo* when it promotes bone repair [30]. Studies also found that a mixture of tooth ash and plaster of Paris with platelet-rich plasma or the fibrin sealant can promote the healing of rabbit skull defects. And platelet-rich plasma has an osteoconduction potential while the fibrin sealant is widely used in hemostasis as well as accelerating the wound healing during bone regeneration [31]. Kim and his teammates have demonstrated that the autogenous tooth bone grafting material had induced active new bone formation and can also gradually be resorbed clinically in the same year. Furthermore, new bone was reconstructed into a more stable bone structure, which owns a noticeable trabecular structure in the human body after 5 months [26].

In this study, calcined tooth powder (CTP) was obtained by a process of 300°C high-temperature burning of teeth for 30 min. This study was designed to clarify the influence of CTP on hDPSCs as well as the involvement of MAPK signaling pathways. Our findings revealed for the first time that calcined tooth powder conditioned medium (CTP-CM) can promote the osteo/odontogenic differentiation of hDPSCs via invoking MAPK signaling pathways. These data provided an important insight into the regulatory effects of CTP-CM on the biological behavior of hDPSCs and provided a theoretical basis for the application of CTP in dentin/pulp tissue regeneration in future endodontic treatment.

## 2. Materials and Methods

### 2.1. Isolation and Culture of hDPSCs

The procedure of cell isolation and culture was performed as described previously [32]. Firstly, clinically healthy and fresh third molars, obtained from normal donors (17-20 years old), were collected in phosphate buffer solution (PBS; Gibco, Grand Island, NY) with the patients' informed consent from the Ethical Committee of Stomatological School of Nanjing Medical University. Teeth were transported to the Ethical Committee of Stomatological School of Nanjing Medical University for cell isolation. The crown of the tooth was split, and pulp tissues were minced and digested with a 3 mg/mL solution of collagenase type I (Sigma, USA) and 4 mg/mL dispase (Sigma, USA) by digestion with PBS in a centrifuge tube for 1 h at 37°C in 5% CO_2_. Followed by centrifugation and resuspension, cells were cultured in alpha-modified Eagle medium (*α*-MEM; Gibco, Grand Island) supplemented with 10% fetal bovine serum (FBS; Gibco, Grand Island), 100 *μ*g/mL streptomycin, and 100 U/mL penicillin (Gibco, Grand Island) at 37°C in a humidified atmosphere containing 5% carbon dioxide. Three days later, the medium was replaced. When cells reached 80% confluence, they were subcultured at a ratio of 1 : 3 by using 0.25% trypsin containing 10 mM EDTA (Sigma, USA). The passage numbers of cells used for this study were limited at 2–5 to avoid cell deterioration.

### 2.2. Preparation of Calcined Tooth Powder and X-Ray Diffraction Observations

The extracted teeth that were without caries, filling, and dental calculus were collected with the patients' informed consent. Any soft tissues attached to the teeth were removed, and then the teeth were dried at room temperature. We annealed the extracted teeth at a temperature of 300°C for 30 min. During this time, the teeth became black and were crushed repeatedly using medical pestles and mortars. The powder was filtered through a 45 *μ*m strainer. The phase compositions of CTP-CM were determined by X-ray diffraction (XRD; SmartLab, Rigaku, Tokyo, Japan). The crystallinity of the tested substances was determined by assessing the width of the base of the XRD peak after scanning (40 kV, 200 mA, scanning rate of 2°/s).

### 2.3. Preparation of Extract of Powder of Calcined Tooth

The calcined tooth powder was added into a-MEM at a concentration of 100 mg/mL and well-mixed and incubated for 1 week at 37°C to obtain the active ingredients of the powder. The supernatant from this preparation was filtered through a 0.22 *μ*m strainer to filter out bacteria before use and then mixed with an equal volume of a-MEM to obtain the CTP conditioned medium. Before using, the CTP conditioned medium was mixed with different volumes of *α*-MEM to required concentrations prior to usage in cell culture experiments [26, 33].

### 2.4. Alkaline Phosphatase Activity Assay

ALP plays an important role during dentin and matrix mineralization. The extract of the CTP conditioned medium was divided into six groups (0 *μ*g/mL, 2 *μ*g/mL, 20 *μ*g/mL, 200 *μ*g/mL, 2 mg/mL, and 20 mg/mL). HDPSCs were seeded into 6-well plates (Corning, USA) and cultured in medium groups with the gradient concentration described above for 5 days. ALP activity assays were performed to select the concentrations with the highest ALP activity by using ALP kits (Nanjing Jiancheng Bioengineering Institute, Nanjing, China) according to the manufacturer's protocol. The experiments were repeated three times.

### 2.5. Alkaline Phosphatase Staining

HDPSCs were seeded into 12-well plates (Corning, USA) and cultured in a complete medium, CTP-CM (200 *μ*g/mL), CTP-CM (200 *μ*g/mL) + SB203580, CTP-CM (200 *μ*g/mL) + SP600125, and CTP-CM (200 *μ*g/mL) + U0126 for 5 days. Cells were washed with PBS for three times. 75% ethyl alcohol was added to fix cells for 30 min at room temperature. The dye solution was prepared according to the alkaline phosphatase staining kit instructions (Bedtime, Nanjing, China). Then, cells were rinsed with deionized water and dyed under dark conditions. The staining results were examined and photographed by scanning (EPSON printer, Japan) and examined under a microscope.

### 2.6. Cell Counting Kit-8 Assay (CCK-8)

Cell proliferation was detected by Cell Counting Kit-8 assay (CCK-8, Dojindo, Japan) according to the manufacturer's instruction. HDPSCs were seeded onto 96-well plates (Corning, USA) at a concentration of 3 × 10^3^ cells/well. Preincubated at 37°C for 24 hours, cells were attached to the wall and then cultured with CTP-CM or complete medium for 9 d. The medium was changed every other day during the incubation period. At days 0, 1, 3, 5, 7, and 9, 10 *μ*L CCK-8 reagent and 90 *μ*L complete medium were added to each well and cells were incubated at 37°C for 2 h. The optical density (OD) was measured at a wavelength of 450 nm by using a microplate reader (Bio-Tek). The experiments were repeated three times.

### 2.7. Flow Cytometry (FCM)

HDPSCs were seeded in 60 mm dishes and cultured in CTP-CM, while cells cultured in complete medium were treated as the control group. Then, cells in the CTP-CM-treated group and the control group were harvested, washed with PBS, fixed with 75% ice-cold ethanol, and stored at 4°C overnight. Afterwards, each sample was washed 3 times with PBS and incubated with propidium iodide (100 mg/mL, Sigma, UK) on ice for at least half an hour in the dark. DNA content was determined by FACScan flow cytometer (BD Biosciences, San Jose, CA). Cell cycle fractions (G0G1/S/G2M phases) were described and compared by flow cytometry. The experiment was repeated three times.

### 2.8. Alizarin Red Staining

HDPSCs were counted and seeded into 12-well multiplates (Corning, USA) at the density of 5 × 10^4^ cells/well. After incubation for 24 hours, the medium was replaced with four different media (complete medium, 200 *μ*g/mL CTP-CM, MM, and MM + 200 *μ*g/mL CTP-CM) for 2 weeks and the media were changed every 3 days. MM is also called mineralization-inducing medium, which was comprised of complete medium (*α*-MEM, 10% FBS, 100 *μ*g/mL streptomycin, and 100 U/mL penicillin), L-ascorbic acid (100 *μ*M, Sigma, UK), *β*-glycerophosphate (10 mM, Sigma, UK), and dexamethasone (10 nM, Sigma, UK). After two weeks, cells were washed with PBS and fixed with 75% precooling ethanol for 30 min. After being rinsed with double-distilled water three times, cells were dyed with 2% Alizarin Red (pH = 4.2, Sigma, UK) at room temperature. The results were examined under a microscope. Photographs were taken in different views. To quantitatively determine the calcified nodules, the Alizarin Red S dye was dissolved by adding 1 mL 10% cetylpyridinium chloride (Sigma, UK) to each well and was shaken for 30 minutes at room temperature. The absorbance at 560 nm was measured using a microplate reader (Bio-Tek). The final calcium levels in two groups were normalized to the total protein concentrations.

### 2.9. Western Blot

HDPSCs were seeded into different 60 mm dishes. Then, cells were treated with the concentration of 200 *μ*g/mL CTP-CM for 3 and 7 days. The cells at the 0 d group were cultured in complete medium without CTP-CM for 7 days [34]. After being washed with PBS for three times, hDPSCs were lysed in RIPA buffer (Bedtime, Nanjing, China) containing 1 mM protease inhibitor phenylmethanesulfonyl (PMSF). To measure the expression of MAPK pathway-related proteins, hDPSCs were, respectively, collected from the protein after cells were treated with the concentration of 200 *μ*g/mL CTP-CM for 0, 15, 30, and 60 min. The cells at the 0 min group were cultured in complete medium without CTP-CM for 60 min. The same dose of phosphatase inhibitor (Bedtime, Nanjing, China) was added to the protein solution compared with PMSF. To validate the impact of inhibitors of MAPK signaling pathways (U0126 targeting ERK, SP600125 targeting JNK, and SB203580 targeting P38), hDPSCs were cultured in serum-free media for 1 d, and then followed by treatment of 200 *μ*g/mL CTP-CM or 200 *μ*g/mL CTP-CM plus 10 *μ*M inhibitors according to the manufacturers' instruction. The total protein was extracted at the specific time points. The same dose of protein was resolved on a 7.5% sodium dodecyl sulfate-polyacrylamide gel electrophoresis (SDS–PAGE) and transferred onto polyvinylidene fluoride (PVDF; Millipore) membranes using a wet transfer apparatus (Bio-Rad, Hercules, USA). The transblotted membranes were blocked in 5% bovine serum albumin (BSA) dissolved in TBST for 2 hours at room temperature and then incubated with indicated primary antibodies for rabbit anti-DSPP (sc-33586, Santa Cruz), rabbit anti-COL-1 (ab34710, Abcam, UK), rabbit anti-ALP (ab95462, Abcam, UK), rabbit anti-RUNX2 (ab76956, Abcam, UK), rabbit anti-OSX (ab22552, Abcam, UK), rabbit anti-OPN (ab8448, Abcam, UK), rabbit anti-OCN (ab93876, Abcam, UK), ERK (#4695, Cell Signaling Technology, USA), p-ERK (#4370, Cell Signaling Technology, USA), JNK (#9252, Cell Signaling Technology, USA), p-JNK (#9255, Cell Signaling Technology, USA), P38 (#8690, Cell Signaling Technology, USA), and p-P38 (#4511, Cell Signaling Technology, USA) in a refrigerator at 4°C overnight. The next day, the membranes were rewarmed at room temperature for 60 min. Then, membranes were washed three times in TBST for 30 min, and TBST was replaced per 10 minutes. After that, membranes were incubated with secondary antibodies (a horseradish peroxidase-conjugated anti-rabbit or anti-mouse IgG) for 60 min. Like the previous steps, membranes were washed for 30 min. At last, the antibody-antigen complexes were visualized with the Western Blot Imaging System (GE Healthcare). The relative protein expression intensities were quantified by densitometry using ImageJ analysis software.

### 2.10. Real-Time Reverse Transcription Polymerase Chain Reaction (Real-Time RT-PCR)

HDPSCs were seeded into dishes with diameters of 60 mm. The culture medium was changed every two days. After 0, 3, and 7 days of culture with CTP-CM, RNA was extracted from cells by using TRIzol reagent (Invitrogen Corporation, USA). The cells at the 0 d group were cultured in complete medium without CTP-CM for 7 days. Extracted RNA was reverse-transcribed to cDNA by MMLV reverse transcriptase (Takara, Tokyo, Japan). Real-time RT-PCR was performed with SYBR Green PCR master mix reagent (Takara, Japan) in an ABI Prism 7300 qPCR system (Advanced Biosystems, USA) as previously described. The samples were subjected to 95°C for 30 s for 1 cycle and 40 cycles at 95°C for 5 s and 60°C for 31 s with specific primers. The expression levels of osteo/odontogenic differentiation markers, including *OCN*, *OPN*, *OSX*, *RUNX2*, *COL-1*, and *DSPP*, were detected with real-time quantitative PCR. Glyceraldehyde-phosphate dehydrogenase (*GAPDH*) was used as the internal reference gene. All samples were run in triplicate. The human gene-specific primers for cDNA can be found in Table 1.

### 2.11. Immunofluorescence Staining

Monolayer hDPSCs were seeded into 24-well plates covered with glass slides and then cultured with complete medium as the control group and CTP-CM for 72 hours. After two washes with PBS, cells were fixed with 4% paraformaldehyde for 30 minutes. Then, hDPSCs were permeabilized with 0.25% Triton-100 for 12 min at room temperature, rinsed three times in PBS, and then blocked with goat serum (DCS/BioGenex, Hamburg, Germany) for 45 min at 37°C. Afterwards, the specimens were incubated with the primary antibodies including DSPP (1 : 100; sc-33586, Santa Cruz), OPN (1 : 100; ab8448, Abcam, UK), and RUNX2 (1 : 100; #8242, Cell Signaling Technology, USA) for 12 hours at 4°C. Subsequently, cells were washed three times and incubated with a secondary antibody labeled with fluorochrome (1 : 50; Invitrogen Corporation, USA) for another 30 min at 37°C in the cassette. Cells were again washed thrice. Nuclei were counterstained with 4,6-diamidino-2-phenylindole (DAPI; 1 : 1000, Sigma, UK) for 90 seconds at 4°C and washed with PBS three times. The coverslips were examined under an inverted fluorescence microscope (Olympus, Japan).

### 2.12. Signal Blocking Assays

U0126, SP600125, and SB203580 (MAPK inhibitors) were purchased from Selleck.cn (http://www.selleck.cn/). HDPSCs were pretreated with the inhibitors for 60 min before stimulation with CTP-CM, while cells were cultured in complete medium as negative control and cultured in CTP-CM as positive control. After 0 min, 15 min, 30 min, and 60 min of stimulation with CTP-CM, the cells were harvested to investigate the inhibition of phosphorylation for several signaling molecules simultaneously. HDPSCs were cultured in complete medium, CTP-CM and CTP-CM with inhibitors. Immunofluorescence analysis was carried out to investigate the protein levels of P-JNK, P-P38, and P-ERK. After 7 days, the osteo/odontogenic differentiation markers OCN, OPN, DSPP, and RUNX2 were evaluated by western blotting and real-time RT-PCR. ALP activity was detected by ALP staining, when cells were cultured with complete medium, CTP-CM, CTP-CM + SB203580, CTP-CM + SP600125, and CTP-CM + U0126 for 5 days. Immunofluorescence analysis was carried out to detect the level of OPN, RUNX2, and DSPP after hDPSCs were cultured in complete medium, CTP-CM, and CTP-CM with inhibitors for 3 days.

### 2.13. Statistical Analysis

Each experiment was performed at least in triplicate, unless otherwise stated. Data were expressed as mean ± SD (standard deviation). The significance of the differences between the experimental and control groups was determined by using one-way analysis of variance. A value of *P* < 0.05 was considered to be statistically significant.

## 3. Results

### 3.1. Morphology Features of hDPSCs and Content Analysis of CTP

**Figure 1:**
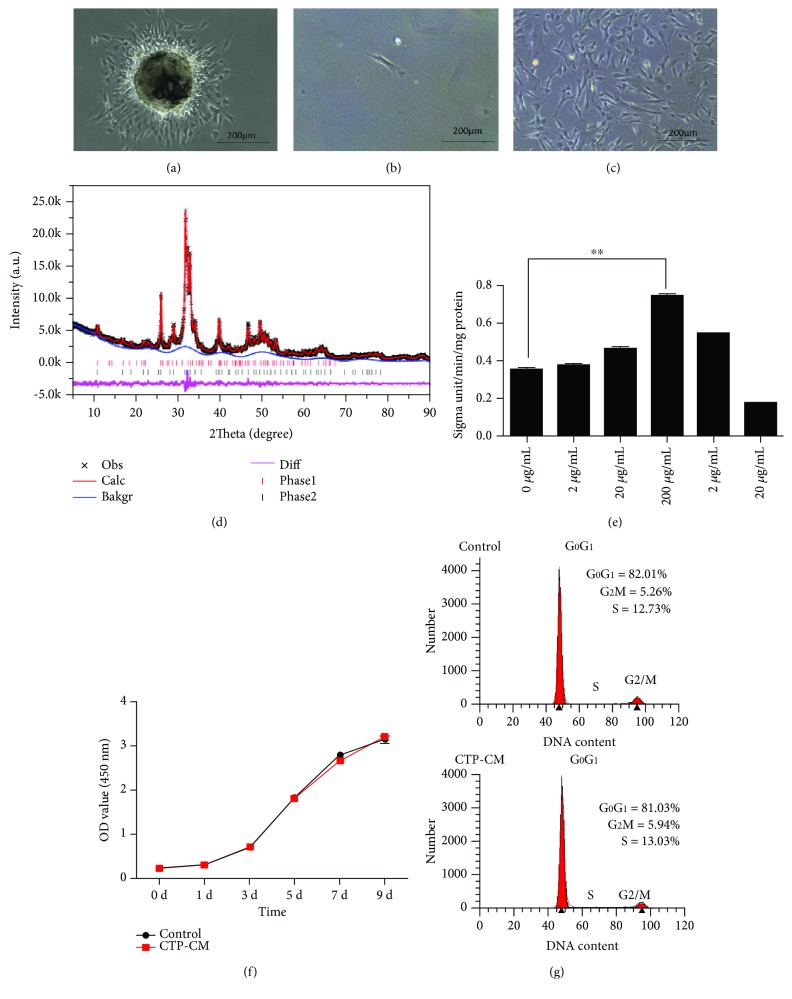
The effects of CTP-CM on the proliferation of hDPSCs. The image of primary hDPSCs was taken after being cultured for 3 days (a). The morphology of a single dental pulp stem cell was of long fusiform or irregular shape (b). The image of hDPSCs in P3 used in all experiments was taken by microscope (c). Scale bar = 200 *μ*m. XRD patterns obtained from CTP (double-headed arrows indicate the base of the peak; *Y* − axis = intensity, *X* − axis = 2*θ*) (d). The ALP activity of hDPSCs treated with conditioned media at different concentrations after 5 days. The graph shows that ALP activity was the strongest at the 200 *μ*g/mL group in this study (e). The proliferation of hDPSCs between the control group and the CTP-CM group was assessed by CCK-8 assay (f) and flow cytometry (g). ^∗^*P* < 0.05 and ^∗∗^*P* < 0.01.

After 3 days of cultivation, primary hDPSC morphology was observed as shown in Figure 1(a). Cells exhibit long fusiform or irregular shape, small size, spiral or radial arrangement, and plastic adherence, similar to the known characteristics of hDPSCs in Figure 1(b). HDPSCs were subcultured to passage 3, which were used at all experiments as shown in Figure 1(c). Further XRD analysis revealed that HA and TCP contents of CTP were 60.75%±4.01% (mean ± SD) and 39.25%±4.01%, respectively, in Figure 1(d).

### 3.2. CTP-CM Did Not Interfere with the Proliferation of hDPSCs

The ALP level is an early marker of osteogenic differentiation. ALP activity assay demonstrated that hDPSCs were cultured with a medium containing 200 *μ*g/mL which presented the highest activity of ALP at all groups in this study (*P* < 0.01, Figure 1(e)), which was selected as the concentration with the best differentiation. CCK-8 assay showed no significant difference in cell growth between the control and 200 *μ*g/mL CTP-CM groups (*P* > 0.05, Figure 1(f)). As indicated by flow cytometry assay, there was no significant difference between the CTP-CM group (PI = 17.99%) and the control group (PI = 19.07%) with respect to the proliferation index (PI = S%+G2M%) (*P* > 0.05, Figure 1(g)).

### 3.3. CTP-CM Promotes Osteo/Odontogenic Differentiation of hDPSCs

**Figure 2:**
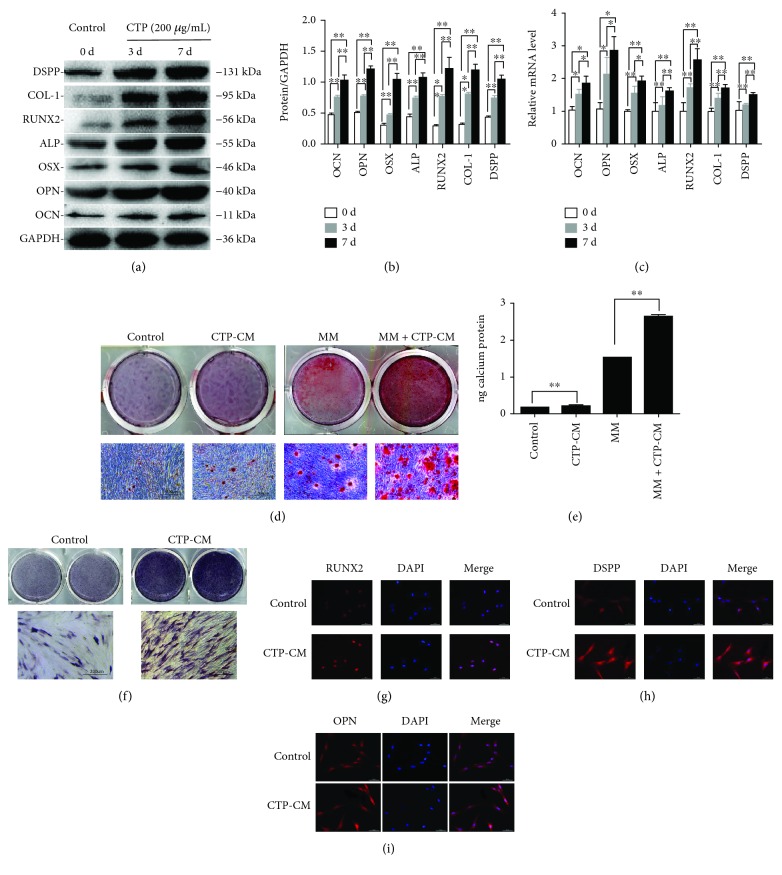
The effects of CTP-CM on osteo/odontogenic differentiation of hDPSCs. Cells were cultured with CTP-CM for up to 0, 3, and 7 days in the concentration of 200 *μ*g/mL. The protein levels were determined by western blot (a). Quantitative analysis of protein bands was done by ImageJ. The data were normalized by GAPDH (b). Total RNA was isolated and analyzed by real time RT-PCR (c), normalized to GAPDH, and quantified relative to the control. Osteogenic differentiation of hDPSCs was assessed by Alizarin Red staining (d) and (e). Images of Alizarin Red S (ARS) staining in hDPSCs in the control group, 200 *μ*g/mL CTP-CM, MM, and MM + 200 *μ*g/mL CTP-CM were scanned directly and taken by a microscope (d). Scale bar = 200 *μ*m. The intensity of Alizarin Red staining was determined by optical density (e). Osteogenic differentiation of hDPSCs was assessed by ALP staining (f). Scale bar = 200 *μ*m. The expressions of DSPP, OPN, and Runx2 in the CTP-CM-treated hDPSC and control groups were detected by immunofluorescence staining (g–i). Scale bar = 50 *μ*m. ^∗^*P* < 0.05 and ^∗∗^*P* < 0.01.

To detect whether CTP-CM could enhance the osteo/odontogenic differentiation of hDPSCs, western blotting, real-time RT-PCR, ALP staining, immunofluorescence staining, Alizarin Red staining, and cetylpyridinium chloride assay were performed. Western blot demonstrated that CTP-CM resulted in the increased expression levels of osteocalcin (OCN), osteopontin (OPN), osterix (OSX), alkaline phosphatase (ALP), runt-related transcription factor 2 (RUNX2), collagen type I (COL-1), and dentin sialophosphoprotein (DSPP) (Figure 2(a)). Furthermore, the protein levels of OCN, OPN, ALP, OSX, RUNX2, COL-1, and DSPP were upgraded significantly in a time-dependent manner at 0, 3, and 7 days, which indicated a promotion of cell differentiation. The result of real-time RT-PCR shows that CTP-CM amplified the levels of gene expression at 0, 3, and 7 days, which was consistent with the above protein expression. Alizarin Red staining showed that the amount of mineralized nodule in the CTP-CM group was higher than that in the control group and CTP-CM + MM had a significantly increased amount of mineralized nodule formation compared with the MM group (Figure 2(d)). Furthermore, cetylpyridinium chloride assay validated that the calcium contents in the control group were lower than those in the CTP-CM group, and the calcium contents in the MM group were significantly less than those in the CTP-CM + MM group (Figure 2(e), *P* < 0.01). The osteogenic differentiation of hDPSCs was initially assessed by measuring alkaline phosphatase (ALP) activity as an early phase marker. ALP staining revealed that CTP-CM enhanced the ALP expression (Figure 2(f)). Immunofluorescence analysis demonstrated that the expression of DSPP, OPN, and RUNX2 in CTP-CM-treated hDPSCs was obviously upregulated than in the control group in both nuclei and cytoplasm (Figures 2(g)–2(i)). The results above imply that CTP-CM plays a critical role during osteo/odontogenic differentiation in hDPSCs.

### 3.4. CTP-CM Induces Activation of MAPK Signaling Pathways during Osteo/Odontogenic Differentiation of hDPSCs

**Figure 3:**
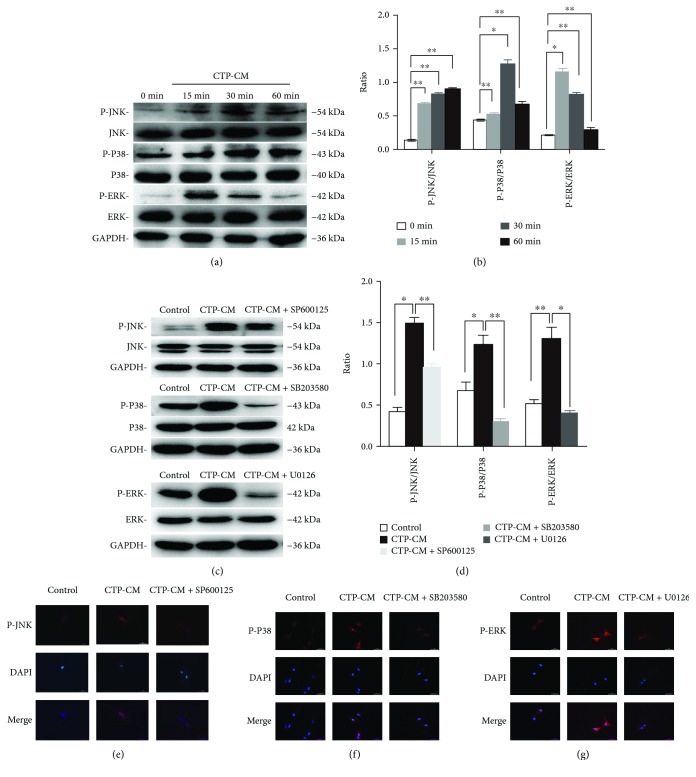
The effects of CTP-CM on MAPK signaling pathways of hDPSCs. All cells were cultured for 60 min, and CTP-CM was added at 60, 30, 15, and 0 min, namely, 0, 15, 30, and 60 min groups. The protein levels of ERK, p-ERK, P38, p-P38, JNK, and p-JNK were determined by western blot (a). Quantitative analysis of protein bands was done by ImageJ. The levels of p-ERK/ERK, p-JNK/JNK, and p-P38/P38 were normalized by GAPDH (b). The hDPSCs were pretreated with MAPK signal inhibitors for 1 hour and then were treated with CTP-CM for the indicated times, when cells cultured in complete medium were treated as the control group. The levels of p-ERK/ERK, p-JNK/JNK, and p-P38/P38 were investigated by western blot (c). Quantitative analysis of p-ERK/ERK, p-JNK/JNK, and p-P38/P38 was performed (d). The protein levels of P-JNK, P-P38, and P-ERK were predicted by immunofluorescence staining (e–g). ^∗^*P* < 0.05 and ^∗∗^*P* < 0.01.

To explore whether MAPK signaling pathways are involved in the CTP-CM-mediated promotion odonto/osteogenic differentiation of hDPSCs, we evaluated the protein levels related to MAPK, such as p-P38, P38, p-ERK, ERK, p-JNK, and JNK by western blotting. Protein levels of p-P38 were upregulated by CTP-CM in a time-dependent manner and reached a maximum at 30 min, then reduced at 60 min. After being cultured with MAPK signaling inhibition SP600125, p-P38 was significantly decreased compared with the 60 min group. Additionally, with CTP-CM treatment, the protein level of p-JNK appeared to rise after 15 min and reached a peak at 30 min, but subsequently decreased after a 60 min treatment. However, the expression level of p-ERK reached a peak at 15 min and then began to decline subsequently (Figures 3(a) and 3(b)). U0126 and SB203580 inhibition exerted a downregulation effect on the protein expression of p-ERK and p-JNK compared with the 60 min group, when cells cultured in complete medium without CTP-CM were treated as the control group (Figures 3(c) and 3(d)). Immunofluorescence staining identifies the expression of P-JNK, P-P38, and P-ERK, showing that the protein levels of P-JNK, P-P38, and P-ERK were downregulated by inhibitors of MAPK signals (U0126 targeting ERK, SP600125 targeting JNK, and SB203580 targeting P38) (Figures 3(e)–3(g)). Therefore, the results indicate that MAPK signals play an important role in CTP-mediated osteo/odontogenic differentiation of hDPSCs.

### 3.5. Inhibition of MAPK Reduces the Osteo/Odontogenic Differentiation of CTP-CM-Treated hDPSCs

**Figure 4:**
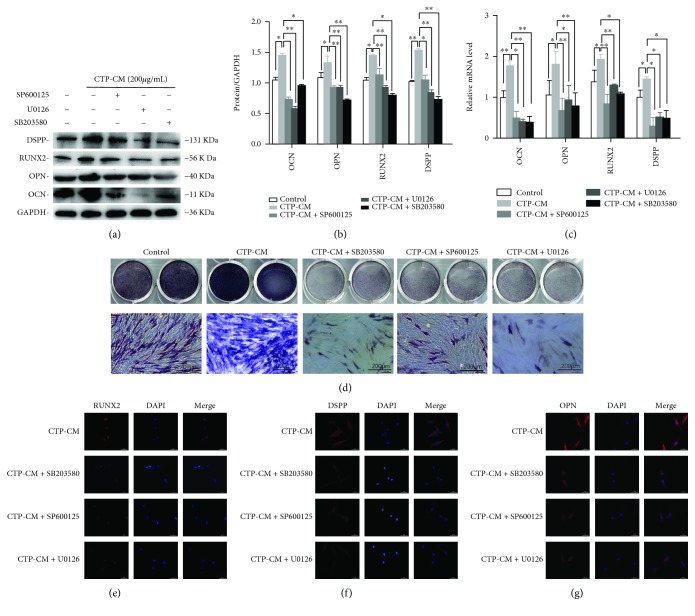
The effects of inhibitors of MAPK signaling pathways on osteo/odontogenic differentiation of CTP-CM-treated hDPSCs. The protein expression of OCN, OPN, RUNX2, and DSPP was analyzed by western blot (a). Quantitative analysis of protein bands was done by ImageJ. The data were normalized by GAPDH (b). The gene expression of odonto/osteogenic differentiation markers *OCN*, *OPN*, *RUNX2*, and *DSPP* were determined by real-time RT-PCR (c). The ALP activity in different groups was detected by ALP staining after the cells were cultured with complete medium, CTP-CM, CTP-CM + SB203580, CTP-CM + SP600125, and CTP-CM + U0126 for 7 days (d). Scale bar = 200 *μ*m. The expression of DSPP, OPN, and RUNX2 in different groups in both nuclei and cytoplasm was revealed by immunofluorescence assay (e–g). Scale bar = 50 *μ*m. ^∗^*P* < 0.05 and ^∗∗^*P* < 0.01.

To further investigate whether the MAPK signaling pathway is involved in the CTP-CM-mediated enhancement of odonto/osteogenic differentiation of hDPSCs, the hDPSCs were cultured in complete medium, CTP-CM, and CTP-CM with inhibitors (U0126, SP600125, and SB203580). After 7 days, western blot and real-time RT-PCR were done to detect the expression of the osteo/odontogenic regulators, including DSPP, RUNX2, OCN, and OPN, which are often used to evaluate the osteo/odontogenic differentiation. The result of western blot demonstrated that CTP-CM can enhance the expression of DSPP, OSX, OCN, and RUNX2, while inhibitors + CTP-CM groups showed significantly lower levels of the odonto/osteoblastic differentiation regulators than did the CTP-CM group (Figures 4(a) and 4(b)). The result of real-time RT-PCR confirmed the result once again (Figure 4(c)). In ALP staining, the ALP level was higher in the CTP-CM group than that in the control group, but the ALP level in CTP-CM + inhibitor (U0126, SP600125, and SB203580) groups was significantly downregulated compared to that in the CTP-CM groups. The results under the microscope were consistent with the above mentioned (Figure 4(d)). The above results were confirmed by immunofluorescence analysis one more time. Immunofluorescence staining identifies the expression of DSPP, OPN, and RUNX2, showing that the protein levels of RUNX2, OPN, and DSPP in groups with inhibitors (U0126, SP600125, and SB203580) inhibit related MAPK signals, and the levels of OPN, RUNX2, and DSPP were decreased compared to the CTP-CM group (Figures 4(e)–4(g)). The result further demonstrates that CTP enhanced osteo/odontogenesis of hDPSCs via MAPK signals.

## 4. Discussion

Nowadays, hDPSCs are being used in clinical work, especially for bone regeneration [35]. Several experiments and studies have shown that hDPSCs are considered as ideal candidates for tissue regeneration with immune rejection [36]. When autologous collection was performed, it has a perfect genetic match [21]. Until now, the specific competence of hDPSCs to form dentin-like structures *in vitro* has been demonstrated in several studies [37, 38]. Responsive to certain biological conditions, DPSCs can develop into odontoblasts, which can secrete the extracellular matrix and initiate mineralization during dentin formation for restoration of pulp vitality and promotion of dental tissue regeneration [39, 40]. HDPSCs have demonstrated the potential to generate dentin and pulp tissues under appropriate environmental conditions *in vitro* [41]. Furthermore, there are also multiple studies that have demonstrated that DPSCs are able to form an unstructured dentin-pulp-like complex with odontoblast-like cells inside when cells were seeded onto a scaffold and transplanted into immunodeficient mice [41]. Due to the high efficiency and high success rate of collection and the excellent interactivity of hDPSCs with exogenous biomaterials for tissue engineering applications, hDPSCs offer good hope and opportunity for future regenerative therapies.

Here we provide evidence that the concentration of 200 *μ*g/mL was selected as the optimal inducing concentration of CTP based on the result of ALP activity. ALP acts as an early-stage marker for calcification, whose expression and activity are increased at the initial phase of mineralization [42]. To explore the effect of CTP-CM on the proliferation capacity of hDPSCs, the cell growth rates were assessed. The results showed that CTP-CM had no effect on cell proliferation. To define expression profiles in hDPSCs treated with CTP-CM, western blot, RT-PCR analysis, ALP staining, Alizarin Red staining, and immunofluorescence staining were conducted. Western blot and RT-PCR analysis revealed that the expression of the osteo/odontogenic markers (OCN, OPN, OSX, ALP, RUNX2, COL-1, and DSPP) by CTP-CM induction for 3 and 7 days was significantly upregulated. Immunofluorescence staining revealed an upregulation of the expression of OCN, OPN, and DSPP protein. Among above osteo/odontogenic markers, DSPP, which acted as odontoblast-specific markers, is associated with the process of dentinogenesis. OPN is also a marker of odontoblast differentiation [36]. RUNX2 and OSX are early markers of osteoblastic differentiation, while OCN mainly appears in the late stages of osteoblastic differentiation [43, 44]. Our results show that CTP-CM promotes an ALP activity level of hDPSCs. Moreover, CTP-CM-treated hDPSCs showed enhanced Alizarin Red staining, which indicates the calcified nodule formation, confirming the inductive effect of CTP-CM in mineralization and differentiation of DPSCs.

Nowadays, vital pulp therapy has become a hot spot in dental regeneration. The main purpose of vital pulp therapy is to preserve the activity and function of residual pulp tissues, to enhance the continuous physiological development of the root, and further to ensure the long-term function of the tooth [45]. The most common method in the clinical treatment is to use pulp capping agents to isolate the external stimuli, clear up the infection and inflammation, and induce hDPSCs to differentiate into osteo/odontoblasts, then form reparative dentin or unstructured hard tissues [46].

The ideal materials used for the vital pulp therapy can induce stem cells to differentiate into odonto/osteoblasts *in vivo* which can form hard tissues, especially the dentin-pulp complex. Besides, the materials must have no immune rejection response. In this study, CTP was obtained from teeth calcined at 300°C high temperature. The organic components of the tooth were completely removed by means of calcining; thus, CTP had no antigenicity. XRD analysis demonstrated that HA accounted for 60.75%±4.01% of CTP and TCP accounted for 39.25%±4.01%. The percentage of TCP in CTP is higher than that in tooth ash obtained in the temperature of 950°C [28]. In direct pulp capping, the agents have close contact with the exposed pulp which is in the moist environment. Researchers have shown that CaP ceramics (HA, TCP, and HA/TCP composite materials) has excellent osteoinductive property and the underlying mechanism is supposed to be the large amount of CaP ions and the chemical reaction of the material with local body fluids which together form a specific microenvironment that induces the migration and osteogenic differentiation of stem cells [21, 36]. Thus, the leaching method was used to prepare CTP-CM in order to simulate the clinical conditions in which the pulp capping agent is located [47].

It has been proved that the multidirectional differentiation of hDPSCs can be regulated by various signaling molecules, receptors, and transcription control systems [29]. Multiple regulatory mechanisms are involved in the process of hDPSC osteo/odontogenic differentiation, in which MAPK signals are included [48]. The MAPK signaling pathway is called intracellular and noncanonical pathway [28]. It is one of the essential signaling molecules which can convert environmental information into a cellular program. The MAPK pathway has been reported to play a key role in cellular responses to a range of stimuli during the process of metabolism, proliferation, and differentiation [42]. Several studies have found the critical function of MAPK signals in regulating the osteogenic differentiation of hDPSCs [32, 46, 49]. P38, JNK, and ERK are the three main members of the MAPK pathway [46]. Under the condition of endogenous or exogenous stimuli, cells responded and different MAPK members as well as their downstream transcription factors were activated [50]. We have further characterized the relevant signaling pathways controlling CTP-induced osteo/odontogenic differentiation of hDPSCs. P38, ERK, and JNK MAPK pathways were immediately activated after hDPSCs were cultured in CTP-CM, while the activation could be partially lowered by corresponding inhibitors, respectively. Meanwhile, the osteo/odontogenic differentiation of hDPSCs was suppressed. These findings demonstrated that CTP-CM positively regulates MAPK pathways and hence potentiate osteo/odontogenic differentiation.

One limitation of this study was that no previous study has investigated the effect of CTP on cellular responses, such as migration, proliferation, and differentiation, as it is a novel material. However, the present results clearly demonstrate that CTP has biocompatibility and nontoxicity to PDLSCs and also encourages their osteo/odontogenic differentiation.

In summary, our study had demonstrated the cellular response of hDPSCs in terms of proliferation and osteo/odontogenic differentiation upon cultivation with CTP. CTP can promote osteo/odontogenic differentiation. Additionally, CTP is less costly and has high reproducibility. Therefore, the present results demonstrate that CTP could be an alternative biomaterial for use in vital pulp therapy, and further studies should be performed *in vivo* to evaluate the effectiveness of this material before its application in future clinical practice.

## 5. Conclusion

The *in vitro* response of hDPSCs to CTP was investigated in terms of proliferation and osteogenic and odontogenic differentiation. In conclusion, the conditioned medium of calcined tooth powder does not interfere with the proliferation of hDPSCs but can promote the osteo/odontogenic differentiation of hDPSCs via triggering MAPK signaling pathways. Furthermore, the activation could be partially lowered by corresponding inhibitors, respectively. Meanwhile, inhibition of MAPK signals can decrease the enhancement of CTP-CM on the osteo/odontogenic differentiation of hDPSCs. It provides a theoretical basis for the directed differentiation of dental pulp stem cells in pulp regeneration.

## Figures and Tables

**Table 1:** Primer sequences for real-time quantitative PCR analysis of gene expression. Sense and antisense primers for real-time reverse transcription plus the polymerase chain reaction (COL-1: collagenase type 1; OPN: osteopontin; ALP: alkaline phosphatase; RUNX2: Runt-related transcription factor 2; OSX: osterix; OCN: osteocalcin: DSPP: dentin sialophosphoprotein; GAPDH: D-gluteraldehyde-3-phosphate dehydrogenase).

Target gene	Sequences (5′-3′)	Product size (bp)	GenBank accession number
*COL-1*	Forward, CTGCAAGAACAGCATTGCAT Reverse, GGCGTGATGGCTTATTTGTT	203	NM_000089
*RUNX2*	Forward, TCTTAGAACAAATTCTGCCCTTT Reverse, TGCTTTGGTCTTGAAATCACA	136	NM_001024630.3
*OSX*	Forward, CCTCCTCAGCTCACCTTCTC Reverse, GTTGGGAGCCCAAATAGAAA	148	NM_001173467.1
*ALP*	Forward, GACCTCCTCGGAAGACACTC Reverse, TGAAGGGCTTCTTGTCTGTG	137	NM_000478.4
*DSPP*	Forward, ATATTGAGGGCTGGAATGGGGA Reverse, TTTGTGGCTCCAGCATTGTCA	136	NM_014208.3
*OPN*	Forward, TGAAACGAGTCAGCTGGATG Reverse, TGAAATTCATGGCTGTGGAA	224	NM_001040060
*OCN*	Forward, AGCAAAGGTGCAGCCTTTGT Reverse, GCGCCTGGGTCTCTTCACT	63	NM_001199662.1
*GAPDH*	Forward, GAAGGTGAAGGTCGGAGTC Reverse, GAGATGGTGATGGGATTTC	225	NM_002046.3

## Data Availability

The data used to support the findings of this study are included within the article.
